# Scum of the Earth: A Hypothesis for Prebiotic Multi-Compartmentalised Environments

**DOI:** 10.3390/life11090976

**Published:** 2021-09-16

**Authors:** Craig Robert Walton, Oliver Shorttle

**Affiliations:** 1 Department of Earth Sciences, University of Cambridge, Cambridge CB2 3EQ, UK; 2 Institute of Astronomy, University of Cambridge, Cambridge CB3 OHA, UK; os258@cam.ac.uk

**Keywords:** prebiotic chemistry, early Earth, origin of life

## Abstract

Compartmentalisation by bioenergetic membranes is a universal feature of life. The eventual compartmentalisation of prebiotic systems is therefore often argued to comprise a key step during the origin of life. Compartments may have been active participants in prebiotic chemistry, concentrating and spatially organising key reactants. However, most prebiotically plausible compartments are leaky or unstable, limiting their utility. Here, we develop a new hypothesis for an origin of life environment that capitalises upon, and mitigates the limitations of, prebiotic compartments: multi-compartmentalised layers in the near surface environment—a ’scum’. Scum-type environments benefit from many of the same ensemble-based advantages as microbial biofilms. In particular, scum layers mediate diffusion with the wider environments, favouring preservation and sharing of early informational molecules, along with the selective concentration of compatible prebiotic compounds. Biofilms are among the earliest traces imprinted by life in the rock record: we contend that prebiotic equivalents of these environments deserve future experimental investigation.

## 1. Introduction

Bulk geochemical environments have several deleterious properties as possible hosts for chemistry that may have given rise to life. Such environments are largely dilute, water-rich, and overly complex—all characteristics that can limit the efficacy of prebiotic reaction pathways. Take the so-called water paradox: the contrast of water as the universal solvent for life with its thermodynamic inhibition of similarly universal condensation reactions involved in, for example, nucleic acid formation [1]. Condensation reactions may be thermodynamically favoured by high concentrations of reactants and a low activity of water. However, high reactant concentrations are often challenging to obtain in dilute bulk solution. This concentration problem is compounded in multi-step reaction paths, whereby a series of linked reversible reactions will produce vanishing small amounts of a desired end-product at equilibrium [2]. Even assuming wholly high-yielding, thermodynamically favourable, irreversible reactions, activation energy barriers to forward reaction may be prohibitive in dilute solutions.

Extant life elegantly overcomes the water paradox, concentration problem, and kinetic barriers by harnessing enzyme-driven reactions to sustain electrochemical gradients across membranes. The cell membrane is built around an amphiphile bilayer that regulates diffusion, with inward facing hydrophobic groups and outward facing hydrophylic groups. This fundamental relationship between spatial structure, energy flow, and chemical behaviour in extant life draws attention to the possible role of structured environments in fostering prebiotic chemistry, which may be more analogous to extant cells than dilute bulk environments, for example, mineral/rock pores, microdroplets, amphiphile-based vesicles, and so on [3,4,5,6]. Here, we propose a novel end-member prebiotic scenario of this kind: near-surface multi-compartmentalised layers of amphiphile-rich material, which we term the scum hypothesis.

## 2. The Scum Hypothesis

The key distinction of our hypothesis from previous protocell-centric scenarios for prebiotic chemistry is environmental in nature. The prebiotic utility of electrochemical gradients across, and low water activity ($a_{w}$) conditions in free-floating proto-cells and amphiphile-rich highly evaporated solutions has been long recognised [5,7,8,9,10,11,12]. In particular, vesicles are known to be valuable for prebiotic chemistry: binding specific reactants and products, initiating chemically productive concentration gradients with respect to the wider environment, and actively participating in key reactions [5,7,10]. However, when isolated, most prebiotically plausible vesicles are leaky, struggling to continuously maintain concentration/free energy gradients sufficient to drive prebiotic chemistry [7]. Essentially, the rate of productive reactions driven by the protocellular environment is outstripped by diffusive and reactive sinks related to the bulk environment.

We suggest that numerous individual compartments may act together to mitigate such deleterious loss of products to the wider environment, thereby aiding prebiotic chemistry. We focus on multi-compartmentalised near-surface environments, scums. Here, an ensemble of leaky compartments achieves the chemical efficacy of individual more functional compartments (with some means of active chemical transport to counter diffusion). We suggest that scum layers may mediate molecular diffusion and water activity to help overcome the concentration problem, arithmetic demon, water paradox, and kinetic barriers to prebiotic chemistry.

### 2.1. Prebiotic Plausibility of Scum Layers

**Figure 1:**
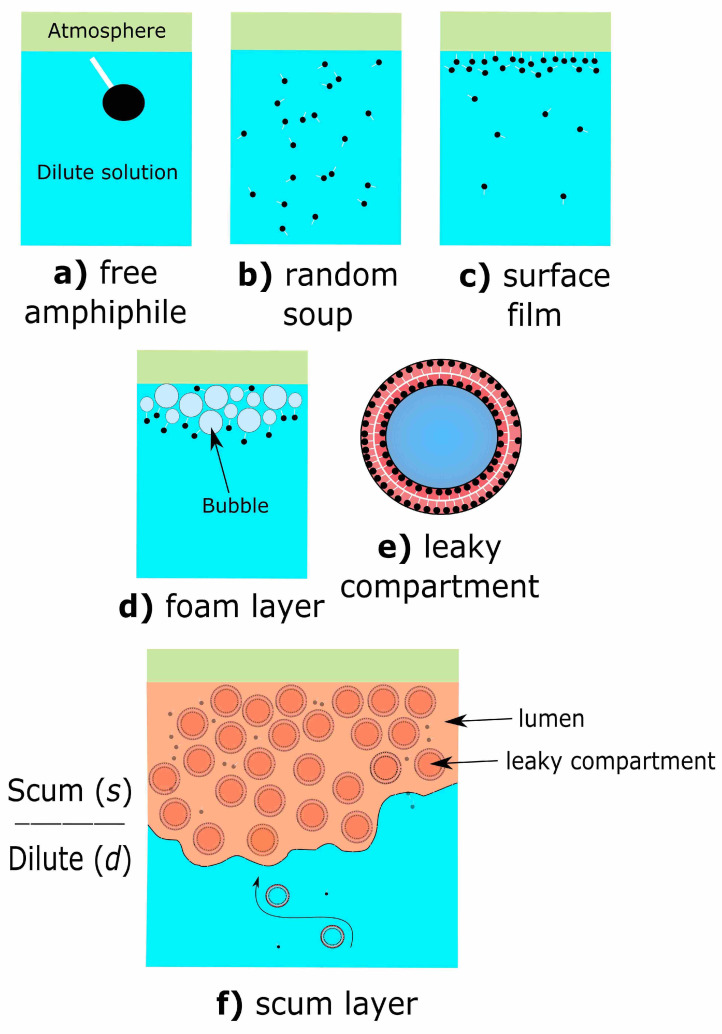
Possible arrangements of simple amphiphiles in a dilute pond. (**a**) Free amphiphile molecule. Black circle represents a hydrophillic head, and the white line represents a hydrophobic tail. (**b**) Zoomed-out view showing randomly oriented amphiphile molecules in an aqueous environment. (**c**) Partitioning of amphiphiles into the near-surface environment, forming a single or multi-layered surface film. (**d**) Foam layer, where amphiphiles are concentrated along complex air–water interfaces created by gas bubbles. (**e**) Generalised amphiphile-based prebiotic compartment, for example, vesicles, consisting of an amphiphilic membrane bilayer. (**f**) Scum layer composed of multiple prebiotic compartments, and an interstial lumen. Thick scum layers may differ noticeably from the wider dilute environment in terms of their overall composition.

Many near-surface environments on Earth, including a large fraction of all lakes and oceans, are characterised by a surface microlayer of distinct physical chemistry and composition to an underlying bulk solution [13]. Amphiphiles may preferentially locate in freshwater and sea surface microlayer due to a lower density of oil solutions compared to bulk water. Elevated surface tension at the air–water interface may also energetically favours amphiphile partitioning into the surface microlayer environment [14,15,16] (Figure 1a). Insolubility and surface-charge adherence can also cause organics to aggregate in the surface microlayer [17]. At high concentrations, these organics can grow into a stable film (Figure 1b,c). However, such organic-rich surface films remain apparently unstructured [17]. Stable aggregates of buoyant organics may be further promoted by the arrival of organic-laden bubbles [17]. Surface foam layers may develop if the air–water interfaces of arriving bubbles are stabilised by surfactant molecules (Figure 1d). Free-floating amphiphile-based structures may form in dilute solutions at sufficient concentrations, forming, for example, vesicles [14,18] (Figure 1e).

Finally, scum layers, the focus of this work, are insoluble organic-rich environments of macroscopic thickness at the water surface (Figure 1f) [19]. Here, we specifically focus on the prebiotic potential of multi-compartmentalised scum layers, consisting predominantly of aggregated vesicles (Figure 1e,f). Vesicles are not the only choice for multi-compartments, but are a plausible candidate. We hypothesise that multi-compartmentalised scum layers may form either by re-organisation of a film or foam into discrete compartments, perhaps in response to changes in bulk composition and/or environmental conditions, for example, wet-dry cycles, or by the surface accumulation of pre-organised structures, for example, buoyant vesicles.

**Figure 2:**
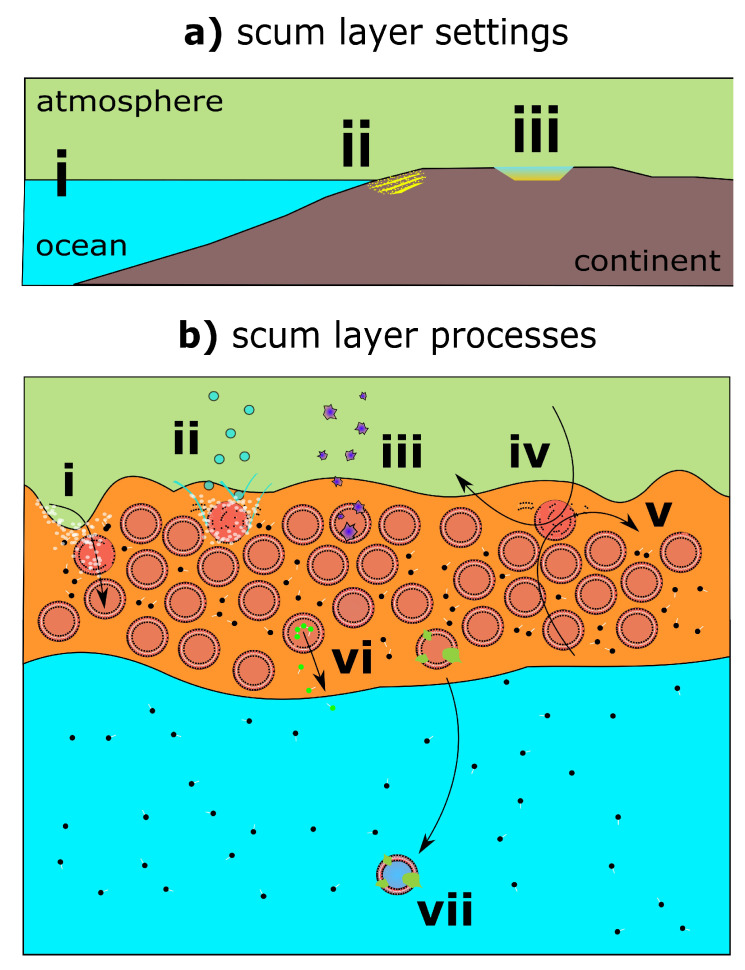
(**a**) Scum layer settings—(i) open ocean, (ii) tidal, (iii) restricted basins. (**b**) Scum layer processes—(i) waves, (ii) precipitation, (iii) cosmic dust settling, (iv) air currents, (v) water currents, (vi) selective molecular diffusion, (vii) mineral precipitation and compartment settling. Disruptive processes are shown intersecting open compartments (red circles, with escaping amphiphiles). White dots represent bubbles. Blue dots represent raindrops. Purple shapes represent cosmic dust grains. Green dots represent ’product’ molecules. Green blobs represent mineral precipitants. Black dots represent free-floating amphiphiles.

There are several early Earth environments where scum layers could have accumulated via these mechanisms. Abiotic amphiphile sources include exogenous fatty acids [20] as well as in-situ synthesis, for example, HCN-centric activation chemistry leading to lipids [21]. Oceans would have largely diluted these sources. However, sea foams do form under agitating conditions in the modern ocean, (speculatively) offering one possible substrate that could have accumulated organics which went on to host multi-compartments (Figure 2a-i). Evaporating saline solutions in tidal zones also represent an opportunity to concentrate amphiphiles (Figure 2a-ii). Only a specific subset of amphiphiles has been demonstrated to self-assemble in saline conditions [22]. These considerations restrict, but do not rule out, the possibility for multi-compartmentalised near-surface scum layers in marine settings.

A body of theoretical, experimental, and field observations suggest that subaerial restricted basins could have accumulated high amphiphile concentrations, which then self-assembled into prebiotic compartments (Figure 2a-iii) [8,23,24,25,26]. Accumulation could have occurred directly at the water surface, owing to the mechanisms described earlier in this paper, or across evaporative cycles [8,25,27]. This latter mechanism has been observed to produce budding rafts of vesicles [25]—perhaps the closest experimentally observed structure to the scum layer that we describe.

Although experimentation and modelling will be needed to test these possibilities, the formation of scum layers on the prebiotic Earth does appear plausible in several environmental settings. Indeed, biologically active multi-compartmentalised scum layers, or biofilms, have been abundant on Earth throughout its history [28]. Such biofilms derive competitive advantages from their ensemble structure [29]—many of which point towards prebiotic utility for prebiotic analogue structures.

### 2.2. Prebiotic Utility of Scum Layers

**Longevity:** The scum hypothesis posits that multi-compartmentalised and buoyant organic-rich environments may have been stronger candidates than free-floating individuals for driving certain reaction pathways in prebiotic chemistry. Much like a modern biofilm, built from constituent cells, a scum layer would have a longer lifespan in aggregate than that of its compartments—and therefore much longer than that of free-floating individuals [29]. Ensemble structures will also be more robust to environmental perturbations than individual compartments [29]. Long-lived scum layers would therefore reduce the chance of destructively dispersing prebiotic molecules into the wider dilute environment.

**Conservation of genetic material:** Conservation of functional/informational polymers would plausibly occur, owing to slower overall diffusion between scum multi-compartments and their bulk environment, than would be observed for isolated compartments (see Section 3 for our quantitative treatment of this important point). By analogy to the behaviour observed in biofilms, compatible molecules released from a collapsed compartment would have their diffusion slowed by, or even be incorporated into, surrounding compartments [29]. Indeed, biofilms are recognised as ideal environments for the sharing of genetic material [29]. Scum layers would therefore similarly have acted to preserve and disseminate informational polymers [30].

**Concentration of prebiotic reactants:** Modern surface scum layers display steep compositional gradients with the underlying bulk solution, evidencing their ability to isolate molecules from dilution. Many surface scum layers on lakes are enriched in particle-bound phosphorus by up to 100-fold versus dilute solution—a process that would appear to be abiotic in nature, related to interfacial binding of particulates [17]. Such processes may be even more effective in high surface area multi-compartmentalised scum layers. Given that high phosphorus-availability is so critical to many proposed reaction pathways, yet thought to be challenging to obtain on prebiotic Earth [31,32,33], the enrichment of phosphorus in near-surface scum layers is of potentially great prebiotic relevance. This process could logically extend to the general capture of aerosols, volcanic ash, and fine-grained exogenous materials (e.g., cosmic dust, spherules; Figure 2b-iii), which could then undergo leaching to contribute essential elements for prebiotic chemistry, which are otherwise typically limiting, for example, P, transition metals, carbon, nitrogen, and sulfur [34].

**Irradiation exposure and attenuation:** Scum layers would have had access to the most intensely ultraviolet (UV)-irradiated regions of an aqueous environment, which increasingly appears to be a strict requirement for performing prebiotic chemistry on near-biological time-spans [35]. Conversely, rapid UV-attenuation by organics in the scum layer could provide a steep irradiation gradient, that is, providing shielding for the products of ongoing prebiotic chemistry [35,36]. The duality of UV exposure and shielding offered by a scum layer may be ideally suited to hosting continuous synthesis pathways that require both UV activity and absence within several reaction steps [33]. Water itself will strongly attenuate UV irradiation intensity within several metres [37]. However, this spatial scale of UV attenuation inherently requires a bulk environment. In contrast, by analogy to organic carbon rich lakes, scum environments may provide shorter-range gradients in both concentration and UV intensity [38]. Meanwhile, the wider environment can serve a different function, both supplying a large reservoir of reactants and removing/diluting those wasteful side products of near-surface chemistry which are not strongly bound to the scum layer.

**Opportunities for environmental cycling:** A viable environmental setting for prebiotic chemistry also requires a mechanism of selective cycling, that is, the earliest stages of evolution by natural selection, wherein individual compartments in the scum represent a form of progenote [39]. This competition-based selection may occur in scum layers in a number of ways. Cycling may occur in restricted basin or tidal scenarios during wet-dry cycles. Universally, scum layers would be prone to disruption during episodic agitation by waves (Figure 2b-i), precipitation (Figure 2b-ii), wind (Figure 2b-iv) or water currents (Figure 2b-v). Length- and charge-dependent diffusion of molecules synthesised in scum compartments into the bulk environment offers a mechanism to select for both certain product molecules and certain types of compartment (Figure 2b-vi). Finally, mineralisation of compartments could cause settling and burial of compartments (Figure 2b-vii). The alternative environment of quiescent ponds would lack many, but not all, of these opportunities for selection. Meanwhile, scum layers in open marine environments could experience any and all forms of disruptive cycling in Figure 2b. However, it is questionable whether these interferences would be too severe, with intense winds and large waves instead entirely disrupting scum layers, or indeed prohibiting their initial stabilisation.

**Caveats:** Standing in the way of these apparent advantages are some important caveats. Modern scum layers are apparently largely unstructured (that is, beyond component films, foams, and biological structures) [17,40]. It therefore remains an open question as to whether prebiotic equivalents would be truly able to self-organise beyond the level of water-air interface structures, and whether near-surface enrichment of, for example, phosphorus can be entirely divorced from ongoing biological activity. The exact degree of UV attenuation by plausible scum layers is also presently unknown, as is their specific ability to concentrate reactants and hold onto products in comparison to isolated vesicles. Overall, there is insufficient evidence at present for positing the scum hypothesis as a holistic scenario for the emergence of life. However, the argument for a potentially constructive role for scum layers in prebiotic chemistry already appears reasonable. In advance of experimental constraints on the detailed prebiotic efficacy of multi-compartmentalised scum layers, we provide first-order calculations to suggest their prebiotic utility by constructing a kinetic model of chemistry taking place in a scum layer.

## 3. A Simple Model of Scum Layer Chemistry

We explore whether scum-type environments have any inherent utility in comparison to isolated compartments for driving prebiotically relevant reactions. Condensation reactions provide a useful case study for these purposes, being ubiquitous in prebiotic chemistry [41]. Take, for example, a generic condensation reaction,

(1) $$\mathrm{COH}+\mathrm{HX}=\mathrm{CX}+H_{2}O.$$

The equilibrium position of this condensation reaction is driven to the right by removal of water, where further forward reaction must occur in order to re-attain equilibrium. Several mechanisms may reduce water activity in naturally occurring prebiotic environments. Molecular crowding in a water-poor solution can result in confining cavities of especially low $a_{w}$, where molecular desolvation can energetically favour condensation [42]. Secondary effects, such as duplex pairing, may emerge to favour condensation reactions involving nucleotides [43,44,45]. These effects are all observed for abiotic nucleotide condensation reactions within a range of water activities from 1–0.01—with confining cavities having much lower water activities in otherwise more dilute bulk solutions [42].

We have previously defined multi-compartmentalised scum layers as buoyant accumulations of structured organic molecules, for example, vesicles and protocells. In addition to further opportunities for the formation of confining spaces, such structured amphiphiles have additional promising properties for driving prebiotic condensation chemistry [46,47,48,49,50,51,52]. Such structures have the potential to actively participate in prebiotic chemistry, or indeed as model protocells, and have already been studied as dispersed populations in this context [5,7].

It is not the intention of this article to prove that scum-type media are specifically more advantageous for driving prebiotic condensation reactions than free-floating prebiotic compartments. However, there are indications that film, foam, and scum layers all have some thermodynamic potential for hosting (at least) prebiotic condensation reactions. With this basic level of prebiotic utility justified, we next explore whether the macroscopic nature of a scum layer may be particularly advantageous for initiating and sustaining dynamic equilibrium—a defining attribute of living cells.

### 3.1. Dynamic Equilibrium: The Drive to Life

Cells must protect biomolecules (e.g., DNA) against degradation by hydrolysis. This protection is ultimately achieved by cycling between the cellular environment and dehydrating microenvironments formed during enzyme-substrate binding [53]. Such continuous cycling results in cell-wide dynamic equilibrium, that is, steady-state, but not thermochemical equilibrium with the wider environment [54]. A key example of dynamic equilibrium is the concentration gradient that universally spans cell membranes. Fluxes between hydrolysis and dehydration in the cell are balanced overall, yet free energy is made available to exploit on local scales via microenvironmental shifts [55]. Without the intervention of enzymes and the bioenergetic membrane, a spatially and temporally homogeneous thermochemical equilibrium would be achieved—corresponding to the cessation of metabolism and other key regulatory activities. A prebiotic system that is incapable of sustaining dynamic equilibria therefore faces a major chemical distinction compared with what we know as life.

The total chemical energy available to do work ($G_{scum}$, J) in the system can be treated as the sum of the products of chemical potentials (μ, $J\;{\mathrm{mol}}^{-1}$), stoichiometric coefficients (*d*), and total moles (*n*) of all species (*i*) undergoing reaction,

(2) $$G_{scum}=\sum_{i}\mu_{i}\;d_{i}\;n_{i}.$$

In systems that reach thermochemical equilibrium, Equation (2) will sum to zero. However, other systems may drive balanced yet continuous fluxes of dehydrated and hydrolysed products into hydrolysing and dehydrating environments, respectively, resulting in non-zero values of $G_{scum}$, that is, thermochemical disequilibrium. At a steady state, this scenario may also be referred to as a dynamic equilibrium.

Consider the case of a selectively leaky prebiotic compartment, which can spontaneously drive forward condensation reaction, owing to, for example, a low internal water activity, and which is also undergoing diffusion-driven exchange with its surroundings. Thermochemical equilibrium in the compartment can only be achieved given either (1) that the compartment is a closed system, which lacks diffusive exchange with dilute surroundings, or (2) the surroundings are non-dilute: product can build up and diffuse back into the compartment at a rate such that an equilibrium position for the reaction is achieved. As the compartment becomes selectively leaky, maintaining the ability to induce forward reaction but more rapidly losing any product generated, a dynamic equilibrium is achieved. Compartments will approach a dilute limit once diffusion removes product as fast as it is generated in. At this dilute limit, the compartment is furthest from being at local thermochemical equilibrium: reactant and product concentrations will reflect those in the wider environment, and Equation (2) will be at a maximum.

A valuable prebiotic environment has the the ability, like life, to exist in between these two states: in a steady-state where thermochemical equilibrium with the wider environment is never achieved, yet a moderate-to-high product concentration is maintained in the compartment in the face of diffusion. This scenario might be most easily found in semi-permeable environments that host exchanges of material between water-poor and water-rich micro-environments, for example, in putative functional semi-permeable protocells, or potentially across multi-compartmentalised scum layers built from individually leaky vesicles. A prebiotic system that recalls extant life is therefore defined by a delicate balance of forward and reverse reaction rates and molecular diffusion, which can be quantified.

### 3.2. A Simple Model of Scum Layer Processes

**Figure 3:**
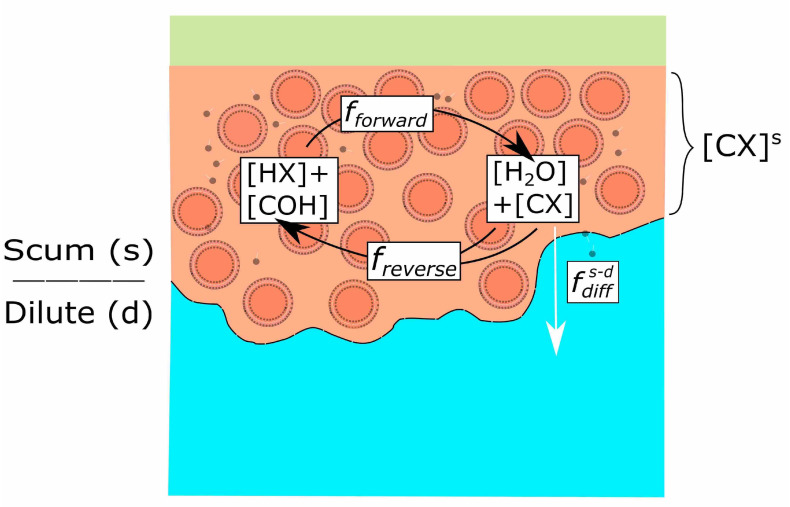
Schematic of fluxes affecting product concentrations in scum environment (s). Reverse reaction flux of product ($f_{reverse}$) is the sum of the flux from reactions occurring in both compartments and interstitial fluid in the scum layer, and therefore varies as a function of total scum volume. Forward reaction ($f_{forward}$) only takes place in compartments. The scum layer also undergoes diffusive loss of product to the wider environment ($f_{diff}^{s-d}$).

We construct a simple kinetic model of condensation chemistry and molecular diffusion taking place in a scum layer (Figure 3). We solve for steady-state. We consider a wider environment (dilute, *d*) and a second multi-compartmentalised environment of prebiotic interest (scum, *s*). The change in concentration of products in environment *s* (Δ[CX], $\mathrm{mol}\;s^{-1}$) must always be zero at a steady state, and can be represented as the balance of product sources and sinks. Sources include forward reaction to form product ($f_{f}^{}$, $\mathrm{mol}\;s^{-1}$). Sinks include reverse reaction of product to form reactants ($f_{r}^{}$, $\mathrm{mol}\;s^{-1}$). Diffusion of product can occur either from scum to dilute environment, or vice versa, and may therefore represent either a source or a sink depending on the direction of diffusion being considered $f_{diff}^{}\left(\mathrm{mol}\;s^{-1}\right)$ (Equation (3)).

(3) $$\Delta {\left[\mathrm{CX}\right]}^{s}=0=f_{f}^{}-f_{r}^{}\pm f_{diff}^{}.$$

Product source and sink fluxes can be written as the product of environmental volumes ($V^{env}$, L) with rates of reaction ($r_{f/r}^{}$, $\mathrm{mol}\;s^{-1}\;L^{-1}$) and diffusion ($r_{diff}^{}$, $\mathrm{mol}\;s^{-1}\;L^{-1}$). We assume that forward reaction 1 (Equation (1)) can only proceed spontaneously in compartments ($V_{c}^{s}$). Reverse reaction can take place in both the compartments and interstitial lumen (Figure 3). A comprehensive treatment of scum diffusion and reaction behaviour is beyond the scope of this article. In order to simplify our model, we consider a selectively leaky scum where reactant concentrations are equal throughout scum and the dilute environment, allowing us to prescribe final reactant concentrations. Diffusive exchange fluxes will depend on the surface areas of contact between scum and dilute environment which, for a fixed thickness of scum, scales with scum volume ($V^{s}$). We can then explore how varying products’ forward and reverse reaction and diffusion rates affect steady-state concentrations of products in scum compartments, writing that (Equation (4))

(4) $$0=r_{f}^{s}\;V_{c}^{s}-r_{r}^{s}\;V^{s}+V^{s}\;\left(r_{diff}^{d-s}-r_{diff}^{s-d}\right).$$

Even though there is no spontaneous forward reaction in environment *d*, there may be diffusion of product from environment *s* into environment *d*. Given that the dilute environment is of much greater volume than the scum layer, we assume that $f_{r}^{d}>>f_{diff}^{s-d}$, such that the concentration of product in in environment *d* is approximately zero at steady state. We can simplify our equation for product sources and sinks in the scum layer accordingly,

(5) $$0=r_{f}^{s}\;V_{c}^{s}-r_{r}^{s}\;V^{s}-f_{diff}^{s-d}.$$

At equilibrium, the rate of product diffusion from environment *s* in our model scenario cannot be greater than the rate of forward reaction, that is, every product molecule produced is balanced via diffusion of a product molecule to environment *d*. In this case we can express product diffusion as some scaled value of the forward reaction rate (Equation (6)),

(6) $$f_{diff}^{s-d}=f_{f}\;K_{diff}.$$

The diffusion scaling constant $K_{diff}$ can vary from 1 (a diffusion flux balancing forward reaction) to 0 (no diffusion from environment *s*). Finally, to obtain the forward and reverse reaction rates, for the general condensation reaction (1), we can write rate laws using model values for forward ($k_{f},\;L\;{\mathrm{mol}}^{-1}\;s^{-1}$) and reverse ($k_{r},\;s^{-1}$) reaction rate constants (Equations (7) and (8)),

(7) $$r_{f}^{}=k_{f}\;\left[\mathrm{COH}\right]\;\left[\mathrm{HX}\right],$$

(8) $$r_{r}^{}=k_{r}\;\left[\mathrm{CX}\right]\;a_{w}.$$

Substituting for unknowns, we can then rearrange Equation (5) and solve for the steady-state concentration of product in the scum layer (Equation (9)),

(9) $$\left[\mathrm{CX}\right]=\frac{k_{f}\;\left[\mathrm{COH}\right]\;\left[\mathrm{HX}\right]\;\frac{V_{c}^{s}}{V^{s}}\;\left(1-K_{diff}\right)}{k_{r}\;a_{w}}.$$

Using this information in conjunction with Equation (2), we can calculate available chemical energy in environment *s*—our measure of whether the equilibrium achieved is more dynamic or thermochemical in nature—as,

(10) $$G_{scum}=\mu_{\mathrm{CX}}\;V_{}^{s}\;\left[\mathrm{CX}\right]+\mu_{\mathrm{HX}}\;V_{}^{s}\;\left[\mathrm{HX}\right]+\mu_{\mathrm{COH}}\;V_{}^{s}\;\left[\mathrm{COH}\right]+\mu_{H_{2}O}\;V_{}^{s}\;\left[H_{2}O\right].$$

We use favorable values for rate constants and values for free energies of reaction that characterise at least some condensation reactions in desolvating (low $a_{w}$) conditions [56,57].

We assume $k_{f}$ = 10 $L\;{\mathrm{mol}}^{-1}\;s^{-1}$, $k_{r}$ = 1 $s^{-1}$, $\mu_{\mathrm{COH}}$ and $\mu_{\mathrm{HX}}$ = 100 $\mathrm{kJ}\;{\mathrm{mol}}^{-1}$, $\mu_{\mathrm{CX}}$ = $-2.5\;\mathrm{kJ}\;{\mathrm{mol}}^{-1}$, $a_{w}$ = 0.1 (assuming open system water loss, with water produced during condensation immediately lost to the environment), [COH] and [HX] = 1 mM, and volume = 1 L in environment *s*. We explore a range of scum layer multi-compartmentalisation densities, from $\frac{V_{c}^{s}}{V^{s}}=0.01$, that is, relatively compartment poor, with a high amount of interstitial fluid, to 0.8, that is, almost entirely dominated by compartments in which forward reaction can spontaneously occur. As water is present on both sides of the generic condensation reaction being considered, water chemical potential is excluded from this calculation.

**Figure 4:**
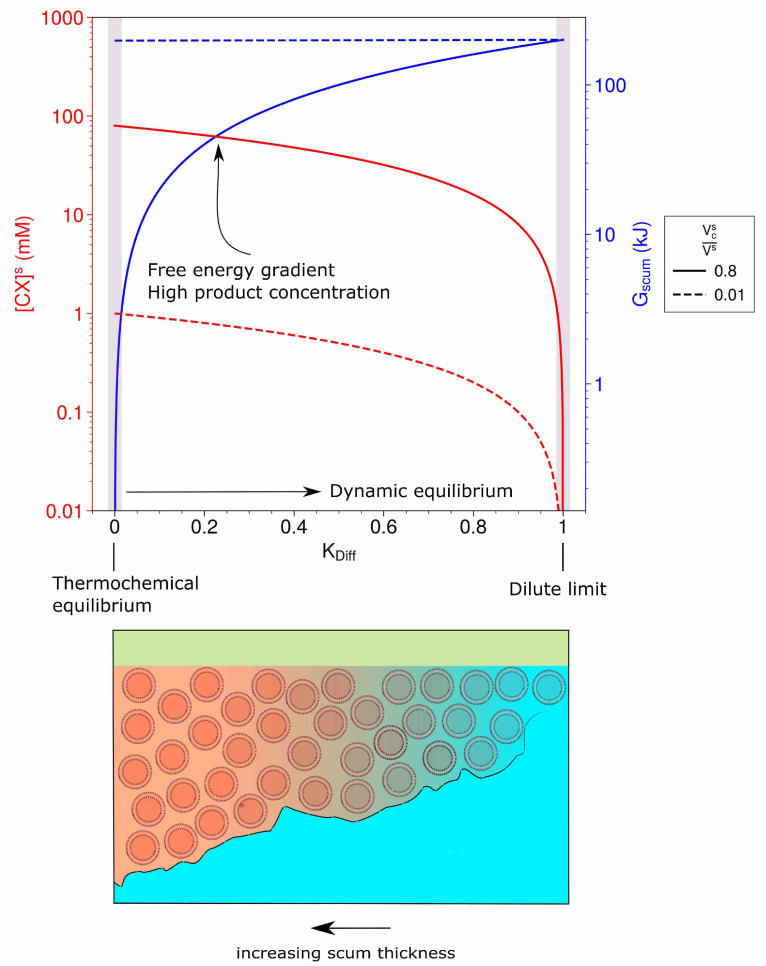
Effect of increasing diffusion scaling constant and reactive compartment volume on equilibrium concentration of product and available energy in environment *s*. Origin scenarios are mapped onto this scale, with both scum layers and functional semi-permeable protocells shown to bridge the gap between fully thermochemical and dynamic equilibrium.

Using these assumptions, we calculate $G_{scum}$ and equilibrium condensation product concentration for a range of diffusion rates between dilute solution (environment *d*) and a scum layer (environment *s*). The results of this calculation are shown in Figure 4. Boundary conditions of thermochemical equilibrium and the dilute limit are explicitly indicated.

If diffusion is rapid enough to remove the majority of product ($K_{Diff}$ greater than 0.5), the dynamic equilibrium approached is one where the free energy available to do chemical work in environment *s* is at its maximum. As the rate of diffusion between environment *s* and *d* is set to lower values (Figure 4), available energy in environment *s* decreases but the equilibrium concentration of product rises. On the other extreme, no diffusion between the two environments will result in thermochemical equilibrium in environment *s*: zero available free energy, but maximum product availability. Maintaining dynamic equilibrium alongside high product concentrations is achievable only given an intermediate diffusion flux from environments s to d. Such a system, which has access to an external environment and yet can maintain concentration gradients against it owing to (thermodynamic and/or kinetic) facilitation of forward reaction, may allow prebiotic reactions to proceed with constant supply—potentially helping to beat universal problems in prebiotic chemistry, such as the arithmetic demon [2].

The diffusion flux of product from a scum to dilute environment depends on factors such as the energy required to cross the interfaces between environment *s* and *d*, the surface area of that interface, the concentration of product in either environment, and the respective volume ratio of the two environments (Figure 4) [58]. Intermediate diffusion fluxes may be observed around a very small environment with a difficult-to-cross interface, for example, a semi-permeable membrane, as in cells or functional protocells, or in a larger environment built from many individually leaky compartments, for example, a multi-compartmentalised scum layer.

The reactive compartment density of the scum layer also plays a critical role in determining behavior, with low ratios of reactive compartment to total scum volume ($\frac{V_{c}^{s}}{V^{s}}$) failing to yield high product concentrations even at thermochemical equilibrium (Figure 4), that is, the system remains detrimentally leaky. This requirement places another constraint on which types of scum layer may have prebiotic utility. However, assuming that leaky compartments are more feasible to assemble on early Earth than biology-adjacent protocells [7], utilising their enhanced aggregate properties via (compartment dense) scum layers would seem to be a viable route for prebiotic chemistry.

Our work shows that free-floating selectively leaky prebiotic compartments exist close to a dilute limit-very far from thermochemical equilibrium, but unable to sustain viable concentration gradients of any product molecules that they may help to spontaneously produce. Meanwhile, multi-compartmentalised scum layers, comprised of functional compartments and interstitial lumen, which together exhibit lower effective rates of diffusion with the dilute environment, provide a possible solution to this dilution problem from the perspective of physical chemistry, deserving of experimental exploration.

## 4. Summary and Next Steps

The origin of life involved linking metabolism, information, and compartmentalisation. In modern cells, bioenergetic membranes maintain the concentration gradients that underlie energy transfer and prevent information from diffusing away. It is possible that fully formed prebiotic analogues emerged with equivalents of these relationships. Alternatively, simpler structures, lacking the ability to actively regulate molecular transport, may have accumulated in near-surface multi-compartmentalised scum environments, benefiting in aggregate from a slower rate of diffusive exchange with the wider environment. We argue that such environments may plausibly have maintained dynamic equilibrium with their environments, without approaching the dilute limit of molecular exchange (Figure 4). In this scenario, dependent upon an external energy source, chemical work is continuously performed, yet useful product concentrations are also maintained against environmental dilution. Scum layers could therefore be a constructive interference with early prebiotic systems, potentially helping to reduce the challenge of regulating molecular diffusion in otherwise dilute prebiotic environments.

The formation of amphiphile-rich film/foam layers and free-floating amphiphile-based structures on prebiotic Earth is already recognised as plausible [8,23,24,25,59]. Multi-compartmentalised scum layers could have emerged on prebiotic Earth via continuous growth and stabilisation of surface films/foams, followed by further self-organisation, or by direct surface/membrane-adherence of pre-formed buoyant structures. Experimental investigation will be required to determine which formation pathways are valid, and which scum types are of prebiotic utility.

Our hypothesis predicts that multi-compartmentalised scum-layers can form during either surface microlayer partitioning or buoyancy-driven aggregation of prebiotically plausible amphiphiles. Experimental work should first establish that prebiotic scum-type structures are plausible, by exploring the physical chemistry of various amphiphile-rich solutions (vesicle-bearing, initially unstructured, and so on) under a range of conditions (quiescence, bubbling, currents, and so on). The formation of unstructured scum layers seems prebiotically plausible on current evidence—the requirements for multi-compartmentalisation, meanwhile, are less obvious. It could be argued that surface-charge interactions may act to repel close-packed prebiotic compartments that are chemically similar to one another. It is therefore possible that distinct membrane types may be required to overcome such electrostatic instabilities, or perhaps mixed systems will also be unstable, leading to unstructured systems of questionable prebiotic utility. We recommend, and intend to pursue, an experimental effort focused on the critical issue of scum formation pathways. If positive results are obtained for prebiotic plausibility of scum layers, future experiments can then begin to consider questions about prebiotic utility.

Scum-type media should be tested for their ability to concentrate reactants from a dilute bulk environment, promote forward reaction, and selectively concentrate product molecules of interest. Results should be compared to those obtained using a mixture of free-floating prebiotic compartments. Important tests include the ability of scum layers to (1) concentrate phosphate and drive phosphorylation reactions, (2) harness a combination of surface scum UV irradiation and juxtaposed UV shielding to drive prebiotic chemistry, (3) drive general condensation polymerisation chemistry, as well as provide selection mechanisms that favour accumulation of longer/more useful products [60], and (4) accumulate and activate fine-grained exogenous materials for use in prebiotic reactions, for example, photocapture by meteoritic PAH pigments/quinone systems, coupled to nucleotide, amino acid, and lipid synthesis pathways [61,62,63,64].

Finally, the ability of scum layers to selectively concentrate molecules of increasing size should be considered. Longer molecules should diffuse more slowly across semi-permeable membranes [65], implying a steady approach towards local thermochemical equilibrium for longer polymers forming in scum-type environments. This behaviour could prove advantageous for maintaining high yields of long chain polymers without needing to invoke a closed system, that is, as may characterised scum environments. It is currently unknown to what extent molecular diffusion rates and forward reaction rates compare between free-floating protocells and scum-type environments. Experimental work is needed to place constraints on these crucial parameters.

Whilst we have pointed to the probable compatibility of scum layers with broadly important condensation reactions, there are many outstanding issues about the diversity, efficiency, and selectivity of prebiotic chemistry taking place in scum layers, as well as the overall placement of these environments in testable scenarios for the origin of life. Despite these outstanding issues, we note that buoyant bacterial communities have a deep ancestry on our planet—representing the earliest known biological structures in the rock record [28]. We contend that prebiotic equivalents of these environments deserve future experimental and theoretical investigation.

## Data Availability

Any further details of the methods or data presented in this paper are available upon request from the corresponding author.
